# Connected Speech Characteristics of Bengali Speakers With Alzheimer's Disease: Evidence for Language-Specific Diagnostic Markers

**DOI:** 10.3389/fnagi.2021.707628

**Published:** 2021-09-07

**Authors:** Arpita Bose, Niladri S. Dash, Samrah Ahmed, Manaswita Dutta, Aparna Dutt, Ranita Nandi, Yesi Cheng, Tina M. D. Mello

**Affiliations:** ^1^School of Psychology and Clinical Language Sciences, University of Reading, Reading, United Kingdom; ^2^Linguistic Research Unit, Indian Statistical Institute, Kolkata, India; ^3^Nuffield Department of Clinical Neurosciences, University of Oxford, Oxford, United Kingdom; ^4^Department of Communication Disorders and Sciences, Rush University, Chicago, IL, United States; ^5^Neuropsychology and Clinical Psychology Unit, Duttanagar Mental Health Centre, Kolkata, India

**Keywords:** speech analysis, Bengali, pronoun, semantic, Alzheimer's disease, connected speech, syntax, micro-linguistics

## Abstract

**Background and aim:** Speech and language characteristics of connected speech provide a valuable tool for identifying, diagnosing and monitoring progression in Alzheimer's Disease (AD). Our knowledge of linguistic features of connected speech in AD is primarily derived from English speakers; very little is known regarding patterns of linguistic deficits in speakers of other languages, such as Bengali. Bengali is a highly inflected pro-drop language from the Indo-Aryan language family. It is the seventh most spoken language in the world, yet to date, no studies have investigated the profile of linguistic impairments in Bengali speakers with AD. The aim of this study was to characterize connected speech production and identify the linguistic features affected in Bengali speakers with AD.

**Methods:** Participants were six Bengali speaking AD patients and eight matched controls from the urban metropolis, Kolkata, India. Narrative samples were elicited in Bengali using the Frog Story. Samples were analyzed using the Quantitative Production Analysis and the Correct Information Unit analyses to quantify six different aspects of speech production: speech rate, structural and syntactic measures, lexical measures, morphological and inflectional measures, semantic measures and measure of spontaneity and fluency disruptions.

**Results and conclusions:** In line with the extant literature from English speakers, the Bengali AD participants demonstrated decreased speech rate, simplicity of sentence forms and structures, and reduced semantic content. Critically, differences with English speakers' literature emerged in the domains of Bengali specific linguistic features, such as the pro-drop nature of Bengali and its inflectional properties of nominal and verbal systems. Bengali AD participants produced fewer pronouns, which is in direct contrast with the overuse of pronouns by English AD participants. No obvious difficulty in producing nominal and verbal inflections was evident. However, differences in the type of noun inflections were evident; these were characterized by simpler inflectional features used by AD speakers. This study represents the first of its kind to characterize connected speech production in Bengali AD participants and is a significant step forward toward the development of language-specific clinical markers in AD. It also provides a framework for cross-linguistic comparisons across structurally distinct and under-explored languages.

## Introduction

Language assessment has a crucial role in the clinical diagnosis of several forms of dementia (Taler and Philips, 2008; Macoir et al., 2015). In Alzheimer's Disease (AD) language has been shown to decline in the pre-symptomatic stages (Snowdon et al., 1996; Ahmed et al., 2013); it is the central feature of primary progressive aphasias (Gorno-Tempini et al., 2011; Grossman, 2012), and acts as a supplementary marker in young onset AD (Crutch et al., 2013). As such, clinical assessment of language has become routine in the diagnostic workup; which commonly use assessment of confrontation naming, verbal fluency; and analysis of spontaneous or connected speech. Connected speech samples elicited *via* picture descriptions, narratives, or interviews have been proven to be better ecological approximations of language production in everyday context. Connected speech goes beyond single-word productions and involves ongoing interactions among diverse cognitive processes including semantic storage and retrieval, executive functions, and memory processes (Ahmed et al., 2013; Mueller et al., 2018; Slegers et al., 2018). Importantly, connected speech samples provide detailed information about processing at several linguistic levels, such as phonetic, phonological, lexico-semantic, syntactic, and discourse-pragmatic; allowing deeper analysis of domains of interest (Boschi et al., 2017).

Recent literature reviews on the linguistic characteristics of connected speech in AD point to a pattern of deficit in several domains including speech rate, syntactic structure and complexity, lexical content, semantic content and efficiency, as well as spontaneity and fluency of speech (Boschi et al., 2017; Mueller et al., 2018; Slegers et al., 2018; Filiou et al., 2020). Specifically, the key features that distinguish AD from healthy control participants are: reduced speech rate and spontaneity including increased repetitions and revisions; simplified syntax and sentence structures including shorter and grammatically simpler sentences; word finding difficulties and increased use of pronouns; inflectional errors in nouns and verbs; and reduced semantic content of speech and uninformative speech with low idea density and efficiency.

With the advantages of quick administration, relatively low burden on the participant, ability to distinguish amongst dementia pathologies, and its use as a marker for disease progression, the evaluation and identification of connected speech characteristics has generated intense interest in dementia research (Gorno-Tempini et al., 2011; Ahmed et al., 2013; Boschi et al., 2017; Mueller et al., 2018; Slegers et al., 2018; Filiou et al., 2020). The progress in the field is encouraging, however, a significant drawback remains with regard to the diversity of languages studied, and how fragmentation of linguistic features differs across different languages (Beveridge and Bak, 2011). Our understanding of linguistic breakdown in dementia is, therefore, limited as the vast majority of studies have been conducted in English speaking participants, with only a few studies in French, Spanish, Brazilian Portuguese, Chinese, Japanese, Hebrew, Iranian, Finnish, Italian, and German (Boschi et al., 2017; Filiou et al., 2020). However, it is well-known from research in language impairments and neurological diseases that language impairments depend on how the system can break down, which in turn is determined by the structure of the language system (Paradis, 1988). For example, syntactic disorders apparent at the surface of a speaker's grammar are dependent on the underlying structure of the specific language. Languages, such as Italian, Spanish, and Bengali are pro-drop languages, that is, they allow speakers to “drop” the subject pronoun if the subject can be inferred from the context. To illustrate, if a Bengali speaker stated, “āmār mā bijñāni” (“My mother is a scientist”), his or her next sentence could be “iunivārsitite kāj karen” (“Works at the university”) in which the pronoun “she” is excluded. Conversely, English, is a non-pro-drop language, that is, speakers must use the subject regardless of the availability of the referent in the context.

This feature becomes all the more important given that one salient marker of language breakdown in AD is the over production of pronouns, such as *he, she, they, it*, rather than use of the specific name or nouns (March et al., 2006; Ahmed et al., 2013; Jarrold et al., 2014; Fraser et al., 2016 for English; Kavé and Levy, 2003; Kavé and Goral, 2016, for Hebrew). However, it remains to be determined if in pro-drop languages individuals with AD would show a similar over production of pronouns or a different pattern might emerge, given that a pronoun is not essential for correct and grammatical production of sentences.

Another feature of note is inflection abilities in AD. Whilst many studies with English speaking AD individuals have shown difficulty with verb inflections in connected speech (e.g., Sajjadi et al., 2012; Ahmed et al., 2013); other studies in English and other languages have not shown difficulty in inflectional morphology for individuals with AD [e.g., Kavé and Levy, 2003; see Auclair-Ouellet (2015) for a review of inflectional morphology in dementia].

There is a critical need to determine language-specific features to accurately describe and understand the linguistic impairments of individuals with AD across different languages. These lines of research will inform assessment procedures, which in turn would lead to more accurate clinical diagnosis of these language users. Compared to English and some European languages, there remains a distinct absence of research evidence documenting the markers associated with language decline in South Asian languages (e.g., Bengali, Urdu, Hindi, Punjabi, Nepalese, and Tamil). The expected growth in neurodegenerative diseases, such as AD will be in low and middle income South Asian and Western Pacific countries including China and India (Prince et al., 2015; Alzheimer's Disease International, 2021). English is not the primary language of use in these countries. Therefore, it is important to identify, characterize, and analyze the linguistic features of connected speech among individuals with dementia from non-English speaking populations. Evaluation of the linguistic profiles of individuals with AD who speak different languages is also key to improve our core theoretical understanding of linguistic impairments across different dementia pathologies. Furthermore, this knowledge has the potential to inform the development and provision of equitable clinical services for the assessment, diagnosis and management for these individuals. The current study fills a significant gap in the research literature and aims to identify and characterize linguistic features of connected speech in Bengali speakers with a clinical diagnosis of AD.

Bengali (also known as Bangla) belongs to the Aryan branch of the Indo-Iranian of the Indo-European group of languages. It is the national language of Bangladesh (first language of 142 million speakers, 98.8% of the total population, Bangladesh Census, 2011) and the official language of three states of India, West Bengal, Tripura, and Assam (first language of 97 million speakers, 8.3% of the total population, India Census, 2011). Bengali is also spoken by the significant global Bengali diaspora (Indian and Bangladeshi) in the United States, the United Kingdom, the Middle East and many Western countries. Bengali is currently ranked as the seventh most spoken language in the world; more than 265 million people speak Bengali as their first or second language in their everyday life. Despite the large number of Bengali speakers there are only handful of studies involving Bengali speakers with neurological impairments (e.g., Lahiri et al., 2019; Patra et al., 2020), and remains one of the under-represented and under-explored world languages in neurological research (Beveridge and Bak, 2011).

In the following section, we highlight the features of Bengali that are relevant for characterization of connected speech production in AD in the domains of syntax, lexicon, and morphology. Table 1 provides a summary of these features and draws attention to the specific differences with English. This table is not intended to include an exhaustive account of all aspects of Bengali, but provides relevant information for characterizing connected speech in the context of AD.

**Table 1:** Summary of relevant linguistic features (syntactic, lexical, and morphology) for Bengali and its contrast with English.

**Syntactic features**	**Bengali**	**English**
Canonical word order	SOV	SVO
Flexibility of word order	Fluid word order at least for canonical forms	Rigid word order for unambiguous sentence construction
Branching	Left branching	Right branching
Passive constructions	Rare to non-existent	Passive constructions are common
**Lexical categories**		
*Open-class words*		
Nouns	Present	Present
Verbs	Present	Present
Compound verbs	Frequent	Infrequent
Adjectives	Present	Present
Adverbs	Present	Present
*Closed-class words*		
Pronouns	Present, pro-drop, similar inflectional system to noun	Present, very limited inflections
Prepositions	Absent	Present
Postpositions	Present	Absent
Auxiliaries	Not present as a word class but represented in the inflectional properties of nouns, verbs, and pronouns	Present
Reduplication	Pervasive usage	Rarely
**Morphological properties**		
*Nominal morphology*	Highly inflected morphology	Limited inflectional morphology
Nouns can be inflected for:		
Number	Marked with suffix	Marked with suffix
Definiteness markers	Marked with suffix	Use of a determiner
Case	Marked with suffix	Not marked
Gender (rarely)	Marked with suffix	Not marked
Particles	Marked with suffix	Not marked
*Verbal morphology*	Highly inflected morphology	Limited inflectional morphology
Auxiliary verbs	Absent	Present
Verbs can be inflected for:		
Tense	Marked with suffix	Marked with suffix
Aspect	Marked with suffix	Marked with auxiliary
Person	Marked with suffix	Marked with suffix but limited
Number	Not marked	Marked with suffix, limited to third person singular
Honorification	Marked with suffix	Not marked
Particles (emphatic and negative)	Marked with suffix	Expressed analytically

To understand the linguistic characteristics of a language, it is useful to consider language typology. It has been shown that word order patterns, such as SOV (Subject Object Verb, in Bengali, Farsi, Hindi, Sanskrit, Latin, and Japanese) or patterns such as SVO (English, Dutch, Italian, Spanish, and Russian) may go hand-in-hand with other language features, such as the existence of pre- or postpositions, the placing of determiners before or after nouns, the presence or absence of pro-drop and of dative subjects, although the clustering of language features is highly complex (Thompson, 2010). Another classifying distinction between languages, which links in with the word order system, is the amount of grammatical inflection. Modern English is predominantly an analytic language, which means that it is made up mainly of free lexical units and there is little remaining inflection. Bengali is a highly inflected language with verbal conjugation according to person, tense, aspect, auxiliary marker, honorification, and particles; and number, particle, and case marking for nouns and pronouns (Dash, 2005, 2015). The inflectional nature of words determines the syntactic roles of the constituents of a sentence. The extent of inflection in a language is usually related to the flexibility of word order. Therefore, in Bengali the SOV order is not mandatory and word order is not rigid. In contrast, English follows a relatively rigid word order.

As mentioned earlier, Bengali is a pro-drop language, allowing omission of personal pronouns in the subject position. Pro-drop occurs in languages with unambiguous conjugational systems where person information is given in the verb inflection. The rules for pro-drop occurrence are context-based. Where the referent is clear from the context, subjects can be dropped. The following are examples of pro-drop sentences produced by participants of this current research:

Example 1:‘tār nām chhila phreṭi’ “His name was Freddy”
‘khub bhālobāsto or dui peṭke’ “Deeply loved his two pets”: Subject dropped
Example 2: ‘maumāchhi tāder tāṛā kare’ “Bees attack them”
‘gāchher guṛite uṭhe paṭe’ “Climb up on a log”: Subject dropped

This pro-drop property of Bengali has important consequences for the amount of pronouns that are produced by speakers in their connected speech.

In terms of lexical distribution, Bengali words belong to seven parts-of-speech: nouns, verbs, adjectives, adverbs, pronouns, postpositions and indeclinables. These grammatical classes can be also organized in terms of open class words (i.e., nouns, verb, adjective, and adverb) and closed class words (i.e., pronoun, postpositions, and indeclinable). Nouns, pronouns, adjectives, verbs and adverbs are inflected in Bengali, whilst indeclinables and postpositions are not.

Bengali nouns are inflected for number, definiteness, gender (rarely), case, and particles. The inflections are tagged in an ordered agglutinative manner to the right side of the nouns to generate the final form.

**Table:** 

Stem	Definiteness	Final Form
din	-ta	dinta
day	-the	the day

**Table:** 

Stem	Plural	Case	Particle	Final Form
din	-guli-	-ke-	-o	dingulikeo
day	-s	-to accusative	Emphatic	to days also

For the inflected noun “*dingulikeo”*, there are three inflections. These three inflections have a fixed order *dingulikeo* (< din + -guli + -ke + -o) and using them in different orders (e.g., < din +-ke + -o+ guli, or < din -o+ +-ke + guli) will generate erroneous forms. Pronouns use a similar set of inflections to nouns.

Bengali verb morphology is extensive and complex, verbs can be inflected for person, tense, aspect, honorification, and particles. In Bengali verbs, person, tense and aspect information are mandatory, whilst honorification and particles can also be added. However, verb inflections do not change with the number and gender of the subject. In contrast to English, Bengali does not have the word classes of auxiliaries, modals, and aspect markers as lexical entities but these are incorporated as inflections on the verbs. To illustrate, the English phrase *He/She/They has/have been writing* is expressed by a single conjugated form /*likhechhe/* in Bengali. Similar to nouns, the inflections are added in a specific order with the verb root to generate the final conjugated form. These conjugated forms generate a complete sense of action as well as aspectual, temporal, and spatial information within the form. Due to the composite nature of inflected Bengali verbs, there is no possibility of dropping a part of an inflection as this will generate an invalid form.

**Table:** 

Root	Auxiliary	Tense	Person	Final Form
dekh	-chh-	-il-	-ām-	dekhchhilām
see	-do	-past	- first person (singular/plural)	I was seeing

**Table:** 

Root	Aspect	Auxiliary	Tense	Person	Final Form
dekh	-e-	-chh-	-il-	-ām-	dekhechhilām
see	-perfect	-do	-past	- first person (singular/plural)	I had done

Bengali has a high occurrence of compound verbs, which is also a prominent feature in many South Asian languages, such as Hindi (Koul, 2008). A compound verb is a two or multiword compound formed by combining a sequence of two or more verbs to act as a single verb to express a single sense or meaning (e.g., *dhare rākh* “catch”, *uṭhe paṛ* “rise”, ś*uye paṛ* “lie down”, *bale phel* “speak”).

In contrast to English, Bengali has fewer word classes within the closed-class category (Bengali: pronouns, postpositions, indeclinables vs. English: prepositions, determiners, pronouns, conjunctions, modals, auxiliaries). Bengali postpositions are similar to prepositions in English. Postpositions occur immediately after a noun or a pronoun to denote spatial, temporal, situational, locational, directional, and conditional information with other words used in a sentence (e.g., *bābār kāchhe* “near father”, *gharer madhye* “in house”, *hāt diye* “by hand”, *dupurer pare* “after noon”, *rāstār dhāre* “beside road”). Akin to English word classes of conjunctions and disjunctions, Bengali has a lexical category collectively known as indeclinables ‘abyay’ which are, in principle, not capable of being inflected (e.g., ā*r* “and”, *ebaṃ* “and”, *bā* “or”, *kintu* “but”, *athabā* “or”).

A frequent feature of Bengali and in many Indian languages is reduplication. Reduplication is a process by which a word is duplicated—wholly or partially—to generate a new word that is different in form and adds new sense in meaning. Reduplication serves multiple semantic functions, such as sense of multiplicity, continuation of action, recurrence of an event or emotional state (e.g., *hāśi* “smile” → *hāśihāśi* “smiling”; *ghuṭ* “dark” → *ghuṭghuṭe* “pitch dark”; → *ghar* “house” *ghar ghar* “in every house”; *din* “day” → *din din* “day by day”). Reduplication can happen to words of all parts-of-speech, although it is more common for open class words.

As can be seen from the above mentioned linguistic features of Bengali, there are distinct differences from English, which can impact manifestation of linguistic impairments in AD. Despite recognition that linguistic impairments are important markers for AD, very little is known regarding patterns of linguistic deficits in speakers of languages other than English. The literature is non-existent with this regard in South Asian languages (e.g., Bengali, Hindi, Urdu, and Punjabi). This research fills a significant gap in the literature and aims to identify linguistic features of connected speech in Bengali speakers with a clinical diagnosis of AD. We used the Frog Story narrative task (“Frog, Where are You?,” Mayer, 1969) to elicit connected speech samples from Bengali AD and matched healthy controls. The multidimensional nature of connected speech and the large number of different variables for analysis that are reported in the literature makes it challenging to decide the best variables to choose to characterize production. The most often used multidimensional analysis framework has been a variant of the Quantitative Production Analysis (QPA; Berndt et al., 2000). In addition, researchers have augmented the QPA with other measures, such as semantic content analysis to capture the semantic breakdown (e.g., Croisile et al., 1996; Ahmed et al., 2013). We implemented and adapted the QPA analysis framework for Bengali as well as used semantic content analysis using the Correct Information Unit analyses (CIU; Nicholas and Brookshire, 1993). As detailed linguistic analysis in Bengali has not yet been reported in connected speech data from neurological impairments, we saw value in covering an exhaustive range of variables in relevant domains to ensure broad range of linguistic features of Bengali are explored. To capture linguistic features specific to Bengali, we supplemented the QPA by adding additional variables (e.g., elaboration of the inflectional morphology for nouns and verbs, inclusion of lexical categories, such as postpositions).

The main objective of the present study was to identify the features of connected speech in the domains of—speech rate, syntactic and grammatical parameters, lexical content, morphological features, semantic content and disruption to fluency and spontaneity—that may be affected in Bengali speakers with AD.

## Materials and Methods

### Ethics Statement

This study was carried out with ethical clearance from the School of Psychology and Clinical Language Sciences, University of Reading (Ref: 2017-035-AB). Participation was voluntary and written consent was obtained from all participants prior to commencement of the study. For participants with AD, consent and information forms were adapted to facilitate comprehension. All participants were able to self-consent to the study.

### Participants

Participants were six right-handed Bengali speaking adults with a clinical diagnosis of AD and eight age-, gender-, education-, and language-matched healthy control participants (HC). Participants were recruited from the Neuropsychology and Clinical Psychology Unit, Duttanagar Mental Health Centre, Kolkata, India. Control participants were recruited from a volunteer participant pool. Exclusion criteria for both groups included a known history of alcohol or drug abuse, or a history of other neurological or psychiatric illness, or <10 years of education.

*Background assessments*. For each participant detailed demographic information was obtained. The level of general cognitive functioning was measured using the adapted Kolkata Cognitive Screening Battery, an adapted Bengali version of Mini-Mental State Examination (BMSE; Das et al., 2006), the Bengali adapted version of Addenbrooke's Cognitive Examination (ACE)-III (Hsieh et al., 2013) and the Clinical Dementia Rating Scale (CDR; Morris, 1993). The CDR is a measure of dementia severity based on the individual's cognitive and daily functions across six domains, which included memory, orientation, judgement and problem solving, community affairs, home and hobbies, and personal care. In addition, the Instrumental Activities of Daily Living Scale for Elderly (IADL-EDR; Mathuranath et al., 2005) assessed patient's ability to undertake day-to-day activities which include cognitive activities (e.g., managing finances, taking medication), social and recreational activities (e.g., looking after grandchildren, pursuing hobbies), community activities (e.g., shopping, travel), household activities (e.g., meal preparation, laundry) and self-care activities (e.g., shaving, personal care). There were 11 items in this scale which were rated for their relevance, levels of impairment, and whether difficulties were caused by cognitive or physical problems. Subsequently, a composite score is derived which indicates the overall physical and cognitive disability. All HC were free of cognitive symptoms or neurological illnesses, and performed within the normal range in KCSB, ACE-III, CDR, and IALD-EDR.

Participants with AD (AD01, AD03, AD04, AD06, AD07, and AD09) were diagnosed by experienced behavioral neurologist and neuropsychologists (fifth and sixth author; AD, RN) using the NINCDS/ADRAA criteria (Mckhann et al., 1984; McKhann et al., 2011). Table 2 provides both AD and HC participants' demographic details and the results of the neuropsychological tests. All participants were Bengali-English sequential bilinguals. They were all native speakers of Bengali and were living in a predominantly Bengali speaking context, using Bengali at home and at work. They were professionally engaged prior to the onset of AD: AD01 was a retired clerk in insurance company; AD03 was a retired electrical supervisor; AD04 managed a farming business; AD06 was a retired tax consultant; AD07 was a homemaker; AD09 was a retired high school teacher. With the exception of AD07 with moderate dementia (i.e., CDR global score of 2), all other AD participants had mild dementia (i.e., CDR global score of 1). At the time of the study, all participants were living with their families in the urban metropolis of Kolkata in eastern India.

**Table 2:** Demographic characteristics and neuropsychological data on the various background measures for each individual with Alzheimer's Disease (AD) as well as Mean and SD of AD and Healthy Controls (HC) groups.

	**Individual cases**						**Group means**						**Results of statistical tests**		
	**AD01**	**AD03**	**AD04**	**AD06**	**AD07**	**AD09**	**Alzheimer's Disease (AD)**		**Healthy Control (HC)**						
							**Mean**	**SD**	**Mean**	**SD**	**Min**	**Max**	***z*** **-value**	***p*** **-value**	**Effect size**
**Demographic information**															
Age at the time of study (years)	67	76	78	51	71	56	66.5	10.89	71.7	4.2	67	78	−0.650	0.516	−0.17
Education (years)	15	14	10	15	17	17	14.7	2.58	16.1	1.2	15	18	−1.088	0.277	−0.29
Duration of symptoms (months)	36	36	24	12	30	48	31.0	12.25							
Age at the onset of symptoms (years)	64	73	76	50	68.5	52	63.9	10.82							
Sex	F	M	M	M	F	F									
Handedness	R	R	R	R	R	R									
**General cognitive functioning**															
Bengali Mini-Mental State Examination^a^ (/30)	22	20	20	22	14	16	19.0	3.29	30.0	0	30	30	−3.441	**0.001**	−0.92
ACE-III, Bengali adapted (/100)^b^	49	40	45	73	27	31	44.2	16.38	92.7	2.3	89	96	−3.102	**0.002**	−0.83
Attention (/18)	11	10	11	13	7	8	10.0	2.19	17.7	0.7	16	18	−3.229	**0.001**	−0.86
Memory (/26)	10	9	12	16	3	4	9.0	4.90	25.3	0.7	24	26	−3.147	**0.002**	−0.84
Fluency (/14)	4	1	0	9	1	1	2.7	3.39	8.0	1.0	7	10	−2.292	**0.022**	−0.61
Language (/26)	16	12	15	24	9	15	15.2	5.04	25.9	0.3	25	26	−3.313	**0.001**	−0.89
Visuoconstructional (/16)	9	8	7	11	7	3	7.5	2.66	15.8	0.4	15	16	−3.233	**0.001**	−0.86
Clinical Dementia Rating (CDR)^c^	1	1	1	1	2	1	1.2	0.41	0.0	0	0	0	−3.528	**0.000**	−0.94
Instrumental Activities of Daily Living Scale in Elderly (IADL-EDR)^d^ (% impairment)	20	50	CNT^e^	11	81	36	39.6	27.56	0.0	0	0	0	−3.338	**0.001**	−0.93

a *Das et al. (2006)*.

b *Hsieh et al. (2013)*.

c *Morris (1993) (CDR score of 0 = no dementia, 0.5 = questionable dementia, 1.0 = mild dementia, 2.0 = moderate dementia, 3 = severe dementia)*.

d *Mathuranath et al. (2005) (a score >16 is in the impaired range with higher value representing higher level of impairment)*.

e *Could not be tested*.

*Bold font in p-values indicate significant difference between HC and AD groups*.

### Experimental Task

A narrative sample in Bengali was elicited using the story book: “Frog, Where Are you?” (Mayer, 1969). Most literature in English speakers with dementias have been elicited using the Cinderella Story retelling narrative task (Kavé et al., 2007; Fraser et al., 2014); whilst the Frog Story has been used by few researchers (e.g., Ash et al., 2007; Ash and Grossman, 2015). For Bengali speakers living in Kolkata, India, it was unlikely that they would know all details of the Cinderella story even if they knew the broad idea of the story. The story of Cinderella is not ingrained in their cultural repertoire as in English speaking or Western countries. We used the Frog Story because we wanted to use a task that would capture relevant and appropriate concepts, and be also culturally acceptable. The stimulus has been successfully used with different types of dementias (Ash et al., 2007).

Prior to administering the narrative task, participants were given a brief background about the story and were told that the main characters of the story are a boy, his dog, and a frog. The story is about a boy who is searching for his missing frog along with his dog. Participants were instructed to look through the picture book and then asked to narrate a story based on the picture book using sentences. Participants could keep the book with them while narrating the story. Tester interruptions were kept to a minimum, other than occasional prompts and generic encouragement. No feedback was provided during the elicitation. Instructions for testing and feedback where written down for the tester to ensure consistency in instruction across participants. The narrative productions were recorded using the digital audio recorder Olympus voice recorder WS-833 for subsequent verbatim orthographic transcription. Excerpts of transcripts from two AD participants (AD03 and AD09) and two HC participants are provided in the Table 3.

**Table 3:** Illustrative samples of the Frog Story narration by two individuals with Alzheimer's Disease (AD) and one Healthy Controls (HC).

**Bengali orthographic transcription**	**Transliteration with Indic Roman**	**English translation**	**Comment**
**AD03** was a 76 year old man who retired several years ago as an electrical supervisor. He had an undergraduate degree with further technical qualifications. He presented to the clinic in Kolkata with a 3 years history of symptoms. He and his family described forgetfulness about meals consumed and the content of recent conversations, difficulty recognizing his own home, and aggression toward family members when in disagreement.			
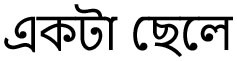	ekṭā chhele	A boy	Utterance, verb missing
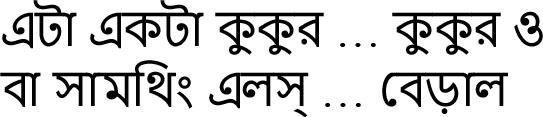	etā ekṭā kukur… kukur o bā something else… beṛāl	This is a dog dog or something else a cat	Repetition and revision
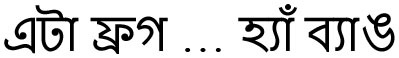	eṭā frog… hyã byāṇ	This frog	Utterance, verb missing, revision
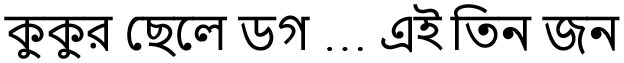	kukur chhele dog … ei tin jan	dog boy dog… these three people	Utterance, verb and predicate missing
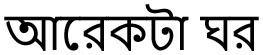	ārekṭā ghar	Another room	Utterance, verb missing
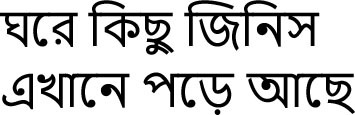	ghare kichhu jinis ekhāne paṭe āchhe	Somethings are scattered here in the room	Correct sentence but unspecific subject
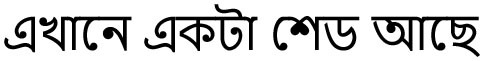	ekhāne ektā śeḍ ācche	There is a shade here	Correct short sentence, use of “āchhe” (i.e., is or has) is of similar pattern to previous construction
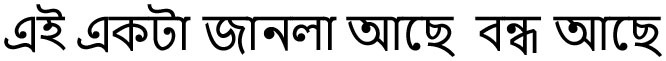	ei ekṭā jānlā ācche bandha āchhe	That is a window… closed	Correct sentence, use of “āchhe” (i.e., is or has) is of similar pattern to previous construction
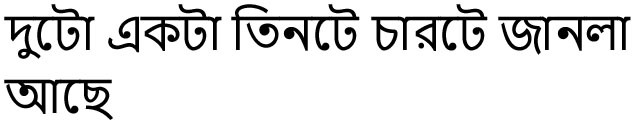	duṭo ektā tinṭe chārṭe jānlā āchhe	Two one three four windows are there	Wrong order of cardinal adjectives, use of “āchhe” (i.e., is or has) is of similar pattern to previous construction
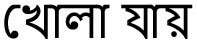	kholā ýāy	Can be opened	Object missing
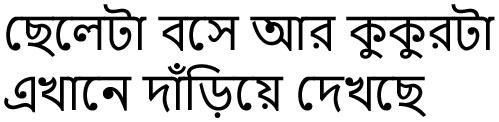	chheleṭā base ār kukurṭā ekhāne dãṭiye dekhchhe	The boy sitting and the dog is seeing standing here	Compound construction but verb missing with the subject in the first noun phrase.
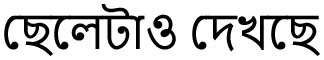	chheleṭāo dekhchhe	The boy is also seeing	Short sentence
**AD09** was a 56 year old woman who took voluntary retirement from her job as a English teacher for high school children following difficulties in coping with the cognitive demands of her job. She had a 4 year history of symptoms including forgetfulness about recent conversations, remembering to convey messages or what she had for meals, as well as remembering what she read. She also experienced word-finding difficulties, and showed increased dependence on her husband for decision making, along with increased topographical difficulties.			
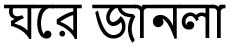	ghare jānlā	Windows in the room	Utterance, verb missing
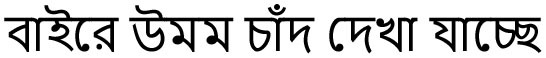	bāire umm chãd dekhā ýāchchhe	Moon is visible outside	Short sentence
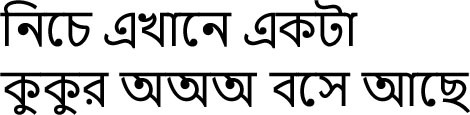	niche ekhāne ekṭā kukur aaa base āchhe	A dog is sitting below	Short sentence
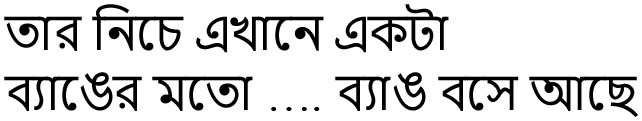	tār niche ekhāne ekṭā byāṛer mato… byāṭ base āchhe	Under this, a frog like frog is sitting there	Revision
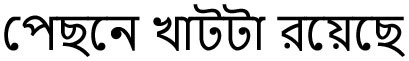	pechhane khāṭṭā rayechhe	The cot is at the back	Short sentence
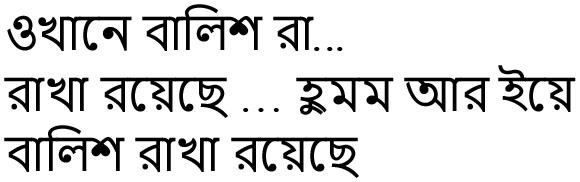	okhāne bāliś rā… rākhā rayechhe… humm ār iye bāliś rākhā rayechhe	There pillow, kept. a pillow is there	Repetitions and revisions, short sentence, use of the same verb token “rayechhe” (is there)
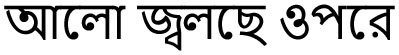	ālo jvalchhe opare	Light is burning at the top	Short sentence
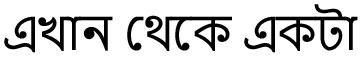	ekhān theke ekṭā	something from there	Vague utterance, subject and predicate missing
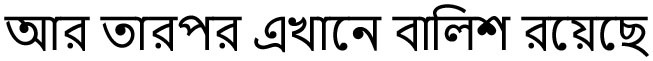	ār tārpar ekhāne bāliś rayechhe	And then after …a pillow is there	Short sentence, use of the same verb token “rayechhe” (is there)
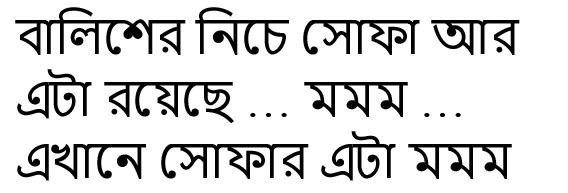	bāliśer niche sophā ār eṭā rayechhe… mmm.ekhāne sophār eṭā mmm	Sofa is under the pillow…is there…here sofa's	Utterance, revisions
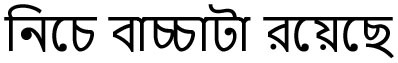	niche bāchchāṭā rayechhe	The boy is under there	Short sentence, use of the same verb token “rayechhe” (is there)
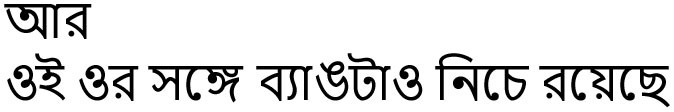	ār oi or saṭge byāṭāo niche rayechhe	and that..The frog is also with him under there	Use of the same verb token “rayechhe” (is there)
**HC09**, 68 year old woman who was a homemaker with 15 years of education (BA degree)			
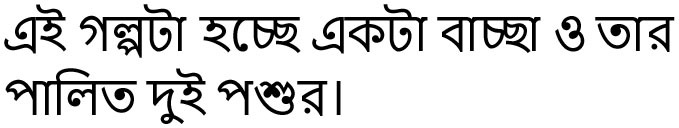	ei galpaṭā hachchhe ekṭā bāchchā o tār pālita dui paśur	This is the story of a boy and his two pet animals.	Coordinated noun phrases, compound sentence.
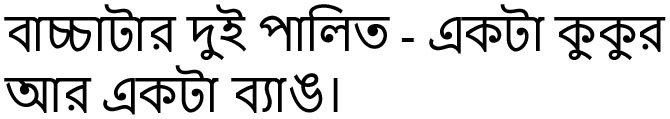	bāchchāṭār dui pālita ekṭā kukur ār ekṭā byāṭ	The boy has two pets, a dog and a frog.	Coordinated noun phrases.
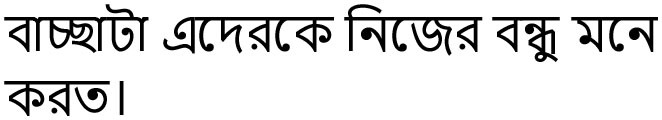	bāchchātā ederke nijer bandhu mane karta	The boy used to think them as his friends.	
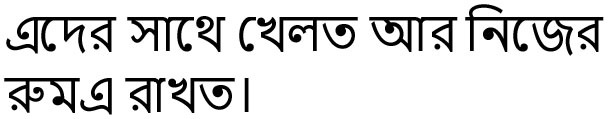	eder sāthe khelta ār nijer rume rākhta	He used to play with them and keep them in his room.	Pro-drop compound sentence
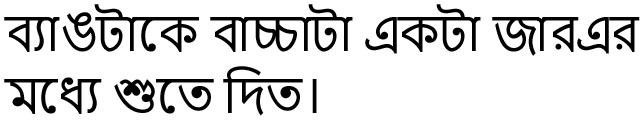	byāṭāke bāchchāṭā ekṭā jārer madhye śute dita	The boy used to keep the frog inside a jar.	
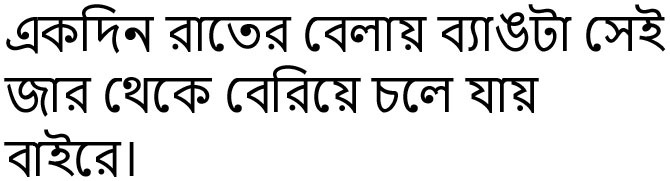	ekdin rāter belāy byāṭā sei jār theke beriye chale ýāy bāire	One night the frog goes out after coming out of the jar.	Flexible word order, postposition at the terminal position. Embedded sentence
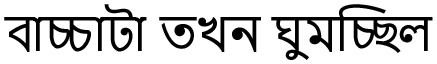	bāchchāṭā takhan ghumachchhila	The boy was sleeping then.	
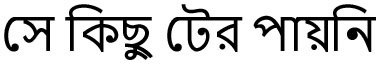	se kichhu ṛer pāyni	He did not know anything (about it).	
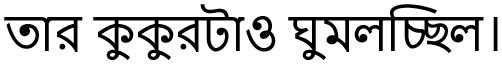	tār kukurṭāo ghumachchhila	His dog was also sleeping.	
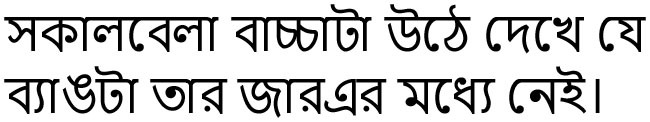	sakālbelā bāchchāṭā uṭhe dekhe ýe byāẹā tār jārer madhye nei	In the morning the boy finds that the frog is not inside the jar.	Embedded sentence
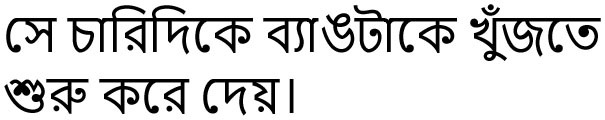	se chāridike byātāke khũjte śuru kare dey	He starts searching for the frog all around him.	Embedded sentence

*The excerpts are the first 12 sentences or utterances from their transcripts*.

### Quantitative Analysis of Narrative Speech

Using the QPA and the CIU analyses we calculated a set of measures for each narrative sample. CIUs are a widely used metric in narrative analysis that assesses the informativeness and efficiency of information conveyed through connected speech (e.g., Carlomagno et al., 2005). The multidimensional nature of connected speech analysis and the large number of different variables used by researchers makes the choosing of appropriate variables to report a challenging task. The measures for this research were in keeping with the recommendations from recent reviews for domains that are essential for characterizing AD speech (Slegers et al., 2018; Filiou et al., 2020). They aimed at quantifying six different aspects of speech production: 1. speech rate; 2. structural and syntactic measures; 3. lexical measures; 4. morphological and inflectional measures; 5. semantic measures (CIU analysis); and 6. measure of spontaneity and fluency disruptions (Wilson et al., 2010; Ahmed et al., 2013; Fraser et al., 2016; Boschi et al., 2017; Slegers et al., 2018; Filiou et al., 2020).

To derive these measures, the narrative samples were transcribed verbatim, segmented and analyzed in accordance with the procedures identical to those used in the QPA (Berndt et al., 2000). As in the original QPA, utterances were defined as segments of running speech that were syntactically and/or prosodically coherent. Placement of sentence boundaries was guided by semantic, syntactic and prosodic features. An utterance did not have to constitute a fully grammatical sentence. Using the QPA rules of extracting the narrative core, words that did not contribute to the narrative were removed, that is, repetitions, repairs, examiner's prompts, discourse markers, non-words (Rochon et al., 2000 for specific steps in extracting the narrative words; please see Berndt et al., 2000). Both the first and second author performed the narrative core extraction individually for all the 14 speech samples. Consensus for any disagreements in narrative core extraction and utterance segmentation were achieved through review of the QPA rules, and re-listening of the audio samples.

The total narrative duration and total number of words produced by each participant were recorded. The minimum length of speech sample for obtaining meaningful results from a narrative production has been widely debated (e.g., Berndt et al., 2000; Sajjadi et al., 2012). The QPA analysis protocol recommends a corpus of 150 words for obtaining meaningful results (Saffran et al., 1989; Berndt et al., 2000). Previous research with different sample lengths have shown that a 150 narrative word corpus produced an adequate and reliable analysis (Sajjadi et al., 2012). To ensure that sample length would not influence the results, we performed our planned analyses using the full sample and ~150-word sample for two AD and two HC participants. The proportional variables on QPA and CIU analyses showed similar, if not identical values for the two sample lengths. Therefore, following recommendation from the literature and to keep the sample length consistent across participants, we derived the measures after extracting 150 ± 10 narrative words.

Using the QPA analysis framework, the narrative samples were analyzed for various measures: structural and syntactic, lexical, and morphological measures (Berndt et al., 2000). Specific linguistic features of Bengali (e.g., postpositions, number of reduplications, number of verbal compounds, verbal, and nominal morphology) were captured by including additional variables to the analysis scheme (see Table 4). We followed the QPA rules for deriving each of these variables; any exception made to the QPA rules to accommodate the characteristics of Bengali is indicated. Semantic content was analyzed using the CIU analyses. The complete list of different variables derived from the analyses is presented in Table 4. The following section provides a brief description of the domains used for characterizing the speech samples between the two groups.

**Table 4:** Summary of the variables that were derived from the narrative production across the six domains of speech production.

	**Linguistic feature**	**Definition/how to measure**
	**Speech Rate**	
	Duration of the narrative (m, sec) sec	The amount of time in the sample containing both speech and pauses. Excluded from the duration were all periods during which the examiner is speaking (Berndt et al., 2000; Rochon et al., 2000)
✓	Total number of words	Total number of words produced by the participants. Indistinct strings of phonemes and discourse markers such as emm, aahh, uuh were excluded from the word count (Rochon et al., 2000; Sajjadi et al., 2012).
✓	Words per minute	Speech rate was defined as the number of words per minute. This measure was calculated on the entire speech sample rather than the 150-word narrative sample that is used to calculate all other measures. We calculated the time from the end of the tester's instructions to the end of participants' production. Number of words was calculated by tallying the total number of uttered words including repetitions, corrections, restarts, and paraphasias as well as patients' direct responses to the questions. Indistinct strings of phonemes and discourse markers such as emm, aahh, uuh were excluded from the word count (Rochon et al., 2000; Sajjadi et al., 2012). Timing and words of the examiner's speech were excluded from the speech rate measure.
	**Structural and syntactic measures**	
✓	Proportion of words in sentences	Total number of words in utterances that were sentences divided total number of sentences.
✓	Mean sentence length	The average number of words produced per sentence.
✓	Proportion well-formed sentence	Total number of well-formed sentences divided by the total number of sentences. As Bengali allows greater flexibility in word order, we recorded the type of errors produced in ill-formed sentences.
✓	Embedding Index	Total number of embeddings divided by the total number of sentences. This measure provides a quantification for utterance complexity. Fewer embeddings would imply less complex utterances.
	**Lexical measures**	
	Number of narrative words (NW)	The number of narrative words were obtained from the transcribed sample after removing habitual starters, stereotype story phrases, examiner's prompts, discourse markers, nonwords, coordinating conjunctions, participants' direct responses to specific questions, comments made by the participant, repetition, and repairs (Berndt et al., 2000). The first 150 ± 10 narrative words were used for the QPA analysis.
	Number of open class words	Sum of all open class words, that is, nouns, verbs, adjectives, and adverbs.
	Number of closed class words	Sum of all closed class words, that is, pronouns, postpositions, and indeclinables.
	Proportion of open class words	Total number of open class words divided by total number of narrative words.
	Proportion of closed class words	Total number of closed class words divided by total number of narrative words.
✓	Proportion of noun (N/NW)	Total number of nouns divided by total number of narrative words.
	Proportion of noun (N/N+V)	Total number of nouns as a proportion of total number of nouns and verbs.
	Noun – verb ratio	Total number of nouns divided by total number of verbs.
	Proportion of noun (N/N+P)	Total number of nouns as a proportion of total number of nouns and pronouns.
✓	Proportion of pronoun (P/NW)	Total number of personal pronouns divided by total number of narrative words.
✓	Proportion of pronoun to noun (P/P+N)	Total number of personal pronouns as a proportion of total number of pronouns and nouns.
✓	Proportion of verb (V/NW)	Total number of verbs divided by total number of narrative words.
	Proportion of verb (V/V+N)	Total number of verbs as proportion of total number of verbs and nouns.
✓	Proportion of non-finite verb (NF/all V)	Total number of non-finite verbs divided by total number of all verbs.
✓	Proportion of matrix verb (MV/all V)	Total number of matrix verbs divided by total number of all verbs.
✓	Proportion of compound verb (CV/all V)	Total number of compound verbs divided by total number of all verbs. This is a Bengali specific characteristic.
	Proportion of adjective (Adj/NW)	Total number of adjectives divided by total number of narrative words.
	Proportion of adverb (Adv/NW)	Total number of adverbs divided by total number of narrative words.
✓	Proportion of postposition (PP/NW)	Total number of postpositions divided by total number of narrative words.
✓	Number of reduplication	Total number of reduplications in the narrative sample. Since the sample size is similar across participants (i.e., ~150 words), the count measure is reported.
	**Morphological and inflectional measures**	
	*Nominal inflections*	
✓	Noun inflection index	Total number of appropriately inflected nouns to the number of nouns that are possible to be inflected. This could be conceptually thought of noun determiner index in English.
✓	Proportion of inflected noun	Total number of inflected nouns to the total number of nouns produced in the narrative.
✓	Proportion of noun with one inflection	Total number of inflected nouns with one inflection to the total number of all inflected nouns.
✓	Proportion of noun with two or more inflections	Total number of inflected nouns with two or more inflections to the total number of all inflected nouns.
✓	Rate of definiteness marker (DM/all N*100)	Total number of nouns inflected with definiteness or number marking to the total number of nouns.
✓	Rate of case markers (CM/all N*100)	Total number of nouns inflected with case marking to the total number of nouns.
✓	Proportion of definiteness marker (DM/N with 1 inflection*100)	Total number of nouns with definiteness marker divided by the total number nouns with single inflections * 100 (e.g., AD04 proportion of definiteness markers 23/35= 65.7%).
✓	Proportion of case markings (CM/N with 1 inflection*100)	Total number of nouns with case markers to the total number of nouns with single inflection*100 (e.g., AD04 proportion of case markers 12/35= 34.3%).
	*Verbal inflections*	
✓	Verb inflection index	Total number of appropriately inflected verbs to the number of verbs that are possible to be inflected. This is conceptually similar to the verb inflection index of the QPA in English.
	Inflection score	It is the sum of total number of tense, aspect and person inflections for the inflected verbs.
✓	Inflection complexity score	It is the ratio of inflection score divided by total number of matrix verb minus 1 (Inflection complexity score=Inflection score/total number of matrix verbs – 1). Inflection complexity score is similar to the auxiliary complexity index in the QPA framework.
	**Semantic measures (CIU analysis)**	
	Word count	To be included in the word count, words had to be accurate, relevant, and informative relative to the eliciting stimuli, and did not have to be used in a grammatically accurate manner (Nicholas and Brookshire, 1993).
✓	Number of CIU	The total number of intelligible, accurate and informative words that were relevant to the Frog story Nicholas and Brookshire, 1993.
✓	Idea density (CIU%)	Total number of CIUs (i.e., semantic units) divided by the total number of words used in the sample.
✓	Idea efficiency (CIUs per minute)	Total number of CIUs (i.e., semantic units) divided by the duration of the sample used for calculation of the CIUs.
	**Measures of spontaneity and fluency disruptions**	
✓	Repetitions	Total number words or whole phrases repeated. For example, whole word (e.g., the < boy> boy was searching for his frog) or phrase-level repetitions (e.g., < The boy>. The boy was searching for his frog). Reduplication of words which is natural phenomenon in Bengali was not considered as repetition (e.g., ā*ste āste* “slowly”).
✓	Revisions	These include when the speaker changes something (usually the syntax) of an utterance but maintains the same idea. It could be word (e.g., a < frog>.dog) or phrase (e.g., < The boy is> …They boy was very upset to not find his frog) revisions.
✓	Reformulations	These included full and complete reformulations of the message without any specific corrections. For example: “ < They boy was searching>.uh he decided to return to the pond".
✓	Total count of disruption of spontaneity	Sum of count of repetitions, revisions and reformulations.

*The check mark (✓) indicates the variables utilized to compare between the groups in this study*.

***Speech rate (words per minute)***. Speech rate was defined as the number of words per minute. That is, the total number of words produced in the narrative divided by the total duration of the narrative.

***Structural and syntactic measures***. This domain measured length, complexity and grammaticality of sentences to capture the structural and syntactic aspects of speech production. Four measures were drawn from various raw structural and syntactic measures (i.e., proportion of words in sentences, mean sentence length, proportion of well-formed sentences, embedding index).

***Lexical measures***. This domain captured subjects' production of various types of lexical items across the entire extracted narrative words, independent of utterance type. These measures included: number of narrative words (NW), number of open class, closed class words, number of nouns (N), verbs (V), compound verbs (CV), non-finite verbs (NF), matrix verbs (MV), adjectives, adverbs, personal pronouns (P), postpositions (PP), and reduplications. A wide range of proportional measures were generated on the basis of these counts of lexical items; full range reported in the Table 4. For this study, we limit reporting and analyzing to a set of variables indicated by check mark () in Table 4. The choice for these variables were motivated by findings in the literature that have been shown to demonstrate dependable differences in connected speech between AD and healthy controls (Slegers et al., 2018; Filiou et al., 2020).

***Morphological and inflectional measures***. To capture the richness and intricacies of the noun and verb inflectional system in Bengali, we generated measures described below. For nominal inflections, we determined the total number of nouns, number of nouns in their base form (i.e., uninflected forms), number of nouns that are possible to be inflected, and number of nouns with appropriate inflections. Additionally, we counted the number of inflections on each noun (i.e., one, two, >two) and the type of those inflections (i.e., definiteness markers vs. case markers, including accusative, genitive, locative). From these count measures, we derived six variables for noun inflections as indicated in Table 4. For verbs, we determined the total number of verbs, number of inflectable verbs, number of inflected verbs with appropriate inflections, and inflection score. From these count measures, verb inflection index and inflection complexity score were calculated to capture inflectional properties of the verbs.

**Semantic measures (CIU analysis)**. Semantic content of the narrative samples was quantified separately using the CIU measures. Words and CIUs were identified from each narrative sample following the procedures outlined by Nicholas and Brookshire (1993). For CIU analysis we used the length of the sample that were used for QPA analysis, rather than the whole sample. Three measures were derived from the CIU analysis: number of CIUs, idea density and idea efficiency.

**Measures of spontaneity and fluency disruptions**. Given that difficulties with fluency and spontaneity have been identified as a salient measure to capture characteristics of AD speech output (Croisile et al., 1996; Ehrlich et al., 1997; De Lira et al., 2011; Slegers et al., 2018), we included a measure called total count of disruption to spontaneity and fluency. This measure included the total number of repetitions, revisions, and reformulations in the narrative sample.

### Statistical Analysis

We approached the analysis in two ways: group and case-series analyses. This is a new set of data in a language that has not been investigated before, thus it is important to capture both group level as well as individual level performance. For the group comparisons, non-parametric versions of independent samples t-test (Mann-Whitney *U*-test) were used for the selected variables. Given the explorative nature of this study and that finding might be informative for under-researched clinical population and potential for future larger scale studies in this area (Perneger, 1998; Feise, 2002), we report findings with exact p-values (both at *p* ≤ 0.01 and *p* ≤ 0.05) and effect sizes for readers to appreciate the strength of these effects. It has been suggested that over-correction of alpha level risks the chance of increasing type II errors (i.e., rejecting significant findings) especially for under-represented clinical populations and hard to recruit populations (Feise, 2002; Streiner, 2009; Streiner and Norman, 2011). Perneger (1998) maintains that over correction leads to a situation where “The likelihood of type II errors is increased, so that truly important differences are deemed non-significant” (p. 1237).

For this research to achieve a balance between Type I and Type II errors (Perneger, 1998; Feise, 2002), and to be erring on caution, we corrected the *p*-value by four (*p* ≤ 0.05/4 = 0.012) for family wise multiple comparisons. The determination of what makes a family for multiple comparison is difficult and ambiguous (Perneger, 1998), especially in a multidimensional phenomenon such as connected speech. The denominator of four is based on the aspects captured by each linguistic domain of the connected speech (i.e., speech rate and spontaneity; structural, syntactic and morphosyntactic measures; lexical measures; and semantic content). Based on the linguistic theories independence across various linguistic domains can be robustly debated, for example, modularity between semantics-syntax, or between semantic-conceptual (Jackendoff, 1972; Caramazza and Zurif, 1976; Moscovitch and Umilta, 1990). Given the inter-correlation of variables amongst linguistic domains, we use four broad domains as family to strike balance between caution and overly conservative interrogation of data.

We implemented Crawford and colleague's single-subject statistical method of comparing a single case to a small control group (at least five) to identify differences between each AD participant and controls (e.g., Crawford and Garthwaite, 2002, 2006; Crawford et al., 2010). This was motivated to facilitate understanding of individual variation and to capture the heterogeneity of the AD population.

## Results

Table 5 provides the mean group data from AD and HC participants; individual data for all six AD participants across different variables; results of group statistics (*p*-values and effect sizes); and results of the single-subject statistics. The readers are encouraged to review Table 3 of illustrative examples of narrative production of AD and HC participants. Table 6 provides the summary of the key findings across the six domains of speech and language production, and information on the proportion of AD individuals who showed similar results to the group differences (i.e., proportion of AD individuals who were significantly different from the controls).

**Table 5:** Individual raw scores for each AD participant, and mean group data from Alzheimer's Disease (AD) and Healthy Controls (HC) across all the connected speech variables along with the results of statistical analysis.

	**Variables**	**Individual AD participants**						**AD group**		**HC group**		**Statistical tests**		
		**AD01**	**AD03**	**AD04**	**AD06**	**AD07**	**AD09**	**Mean**	**SD**	**Mean**	**SD**	***z*** **-value**	***p*** **-value**	**Effect size**
	**Speech rate**													
	Duration of the narrative, sec (s)	269	509	466	294	87	764	398.20	234.60	201.13	50.90	−1.94	0.053	−0.52
✓	Total number of words	320	406	229	276	164	537	322.00	133.43	466.00	211.98	−1.42	0.156	−0.38
✓	Words per minute	71.3	48.2	29.35	56.3	113	42.2	60.07	29.52	135.92	31.89	−2.97	**0.003**	−0.79
	**Structural and syntactic measures**													
	Number of sentences	38	32	34	23	15	38	30.00	9.19	19.63	2.83	−1.94	0.052	−0.52
	Number of topic/comment utterances	4	9	6	5	4	11	6.50	2.88	2.63	2.26	−2.21	**0.027**	−0.59
	Number of embeddings	1	0	0	1	2	0	0.67	0.82	9.38	2.56	−3.12	**0.002**	−0.83
	Number of well-formed sentences	28	28	28	13	13	33	23.83	8.61	18.13	3.14	−1.05	0.294	−0.28
	Number of words in sentence	148	138	138	120	68	127	123.17	28.72	147.50	12.20	−1.94	0.052	−0.52
	Number of words in topic or comments	12	29	21	18	14	32	21.00	8.05	10.38	10.50	−1.88	0.061	−0.50
✓	Proportion of words in sentences	0.93	0.83	0.87	0.87	0.84	0.80	0.85	0.04	0.93	0.07	−1.94	0.052	−0.52
✓	Mean sentence length	3.89	4.31	4.06	5.22	4.53	3.34	4.23	0.63	7.59	0.73	−3.10	**0.002**	−0.83
✓	Proportion of well-formed sentences	0.74	0.88	0.82	0.57	0.87	0.87	0.79	0.12	0.92	0.07	−2.53	**0.011**	−0.68
✓	Embedding index	0.03	0.00	0.00	0.04	0.13	0.00	0.03	0.05	0.50	0.18	−3.11	**0.002**	−0.83
	**Lexical measures**													
	Number of narrative words (NW)	160	167	159	138	81	159	144.00	32.37	158.00	6.00	−0.26	0.795	−0.07
	Number of open class words	130	131	126	109	69	136	116.83	25.20	120.00	6.99	−0.65	0.516	−0.17
	Number of closed class words	30	36	33	29	12	23	27.17	8.61	38.00	7.15	−2.13	**0.033**	−0.57
	Proportion of open class word	0.81	0.78	0.79	0.79	0.85	0.86	0.81	0.03	0.76	0.04	−2.14	**0.033**	−0.57
	Proportion of closed class words	0.19	0.22	0.21	0.21	0.15	0.14	0.19	0.03	0.24	0.04	−2.14	**0.033**	−0.57
	Number of nouns (N)	62	52	61	41	23	51	48.33	14.58	52.75	4.62	−0.32	0.746	−0.09
	Number of verbs (V)	43	41	42	39	20	45	38.33	9.20	37.38	6.14	−0.84	0.40	−0.23
	Number of nonfinite verbs (NF)	9	9	8	15	3	4	8.00	4.29	14.25	3.37	−2.14	0.033	−0.57
	Number of matrix verbs (MV)	33	32	34	24	17	41	30.17	8.42	23.13	4.42	−1.82	0.069	−0.49
	Number of compound verbs (CV)	22	9	13	11	9	15	13.17	4.92	12.25	4.13	−0.07	0.948	−0.02
	Number of adjectives (Adj)	2	30	8	9	10	19.00	13.00	9.96	14.25	2.92	−0.84	0.401	−0.22
	Number of adverbs (Adv)	5	5	3	6	4	7.00	5.00	1.41	4.50	3.25	−1.18	0.238	−0.32
	Number of all pronouns	5	11	13	17	6	7.00	9.83	4.67	19.25	5.26	−2.59	**0.010**	−0.69
	Number of demonstrative pronouns	1	7	5	1	1	1.00	2.67	2.66	2.88	1.73	−0.66	0.510	−0.18
	Number of personal pronouns (P)	4	4	8	16	5	6	7.17	4.58	16.38	4.66	−2.66	**0.008**	−0.71
	Number of postpositions (PP)	20	10	15	12	6	19.00	13.67	5.39	12.13	3.91	−0.78	0.438	−0.21
✓	Number of reduplication	0	0	1	1	1	0	0.50	0.55	3.00	2.78	−1.99	**0.046**	−0.53
	**Proportional measures from lexical counts**													
✓	Proportion of noun (N/all NW)	0.39	0.31	0.38	0.30	0.28	0.32	0.33	0.04	0.33	0.03	−0.71	0.476	−0.19
	Proportion of noun (N/N+V)	0.59	0.56	0.59	0.51	0.53	0.53	0.55	0.03	0.59	0.04	−1.57	0.118	−0.42
	Noun – verb ratio: #N/#V	1.44	1.27	1.45	1.05	1.15	1.13	1.25	0.17	1.44	0.21	−1.43	0.154	−0.38
	Proportion of noun (N/N+P)	0.94	0.93	0.88	0.72	0.82	0.89	0.86	0.08	0.76	0.06	−2.13	**0.033**	−0.57
✓	Proportion of pronoun (P/all NW)	0.03	0.02	0.05	0.12	0.06	0.04	0.05	0.03	0.10	0.03	−2.27	**0.023**	−0.61
✓	Proportion of pronoun to noun (P/P+N)	0.06	0.07	0.12	0.28	0.18	0.11	0.14	0.08	0.24	0.06	−2.13	**0.033**	−0.57
✓	Proportion of verb (V/all NW)	0.27	0.25	0.26	0.28	0.25	0.28	0.27	0.02	0.24	0.04	−1.43	0.152	−0.38
	Proportion of verb (V/V+N)	0.41	0.44	0.41	0.49	0.47	0.47	0.45	0.03	0.41	0.04	−1.57	0.118	−0.42
✓	Proportion of nonfinite verb (NF/all V)	0.21	0.22	0.19	0.38	0.15	0.09	0.21	0.10	0.38	0.07	−2.79	**0.005**	−0.75
✓	Porportion of matrix verb (MV/all V)	0.77	0.78	0.81	0.62	0.85	0.91	0.79	0.10	0.62	0.07	−2.73	**0.006**	−0.73
✓	Proportion of compound verb (CV/all V)	0.51	0.22	0.31	0.28	0.45	0.33	0.35	0.11	0.34	0.12	−0.26	0.796	−0.07
	Proportion of adjective (Adj/NW)	0.01	0.18	0.05	0.07	0.12	0.12	0.09	0.06	0.09	0.02	−0.13	0.897	−0.04
	Proportion of adverb (Adv/NW)	0.03	0.03	0.02	0.04	0.05	0.04	0.04	0.01	0.03	0.02	−1.18	0.236	−0.32
✓	Proportion of postposition (PP/NW)	0.13	0.06	0.09	0.09	0.07	0.12	0.09	0.03	0.08	0.02	−1.31	0.192	−0.35
	**Morphological and inflectional measures**													
	***Nouns inflections***													
	Total number of nouns	62	52	61	41	23	51	48.33	14.58	52.75	4.62	−0.32	0.746	−0.09
	Number of nouns in base form	14	29	15	19	7	25	18.17	7.96	22.38	6.67	−1.18	0.240	−0.31
	Number of nouns possible to be inflected	48	23	45	22.00	16	26	30.00	13.22	30.38	4.47	−0.91	0.364	−0.24
	Appropriate noun inflection	47	23	45	22.00	16	24	29.50	13.10	30.38	4.47	−0.97	0.330	−0.26
✓	Noun inflection index	0.98	1.00	1.00	1.00	1.00	0.92	0.98	0.03	1.00	0.00	−1.70	0.090	−0.45
	*Noun inflection type*													0.00
	Total number of inflected nouns	48	23	45	22	16	24	29.67	13.37	30.38	4.47	−0.97	0.330	−0.26
	N with 1 inflection	36	18	35	18	14	22	23.83	9.39	25.50	3.07	−0.91	0.364	−0.24
	N with 2 inflections	10	5	10	4	2	2	5.50	3.67	5.57	2.94	0.00	1.000	0.00
	N with >2 inflections													0.00
✓	Proportion of inflected nouns	77.4	44.2	73.8	53.7	69.6	47.1	60.95	14.39	58.05	10.72	−0.26	0.796	−0.07
✓	Proportion of noun with 1 inflection	0.75	0.78	0.78	0.82	0.88	0.92	0.82	0.06	0.85	0.09	−0.58	0.559	−0.16
✓	Proportion of noun with 2 or > inflections	0.21	0.22	0.22	0.18	0.13	0.08	0.17	0.06	0.18	0.07	−0.39	0.697	−0.10
	Inflection type: Definiteness marker (DM)	24	14	23	5	8	16	14.83	7.96	6.88	2.85	−1.88	0.060	−0.50
	Inflection type: Case markers (CM)	12	4	12	13	6	6	8.83	3.54	18.50	3.89	−2.79	**0.005**	−0.75
	Rate of Definiteness marker (DM/all N*100)	38.7	26.9	37.7	9.8	34.8	31.4	29.87	10.76	13.08	5.55	−2.45	**0.014**	−0.66
	Rate of case markers (CM/all N*100)	17.7	9.6	19.7	31.7	26.1	11.8	19.43	8.40	35.11	6.87	−2.84	**0.005**	−0.76
✓	Proportion of Definiteness marker (DM/N with 1 inflection*100)	66.7	77.8	65.7	22.2	57.1	72.7	60.38	19.95	27.09	12.07	−2.45	**0.014**	−0.66
✓	Proportion of case markings (CM/N with 1 inflection*100)	30.56	27.78	34.29	72.22	42.86	27.27	39.16	17.18	72.44	12.56	−2.71	**0.007**	−0.72
	***Verb inflections***													
	Number of verbs	43	41	42	39	20	45	38.33	9.20	37.38	6.14	−0.84	0.40	−0.23
	Number of inflectable verbs	43	41	42	39	20	45	38.33	9.20	37.38	6.14	−0.84	0.40	−0.23
	Number of inflectable verbs inflected	43	41	42	39	20	45	38.33	9.20	37.38	6.14	−0.84	0.40	−0.23
✓	Verb inflection index	1	1	1	1	1	1	1.00	0.00	1.00	0.00	0.00	1	0.00
	Inflection score (IS)	98	96	102	72	51	123	90.33	25.21	69.00	12.63	−1.75	0.081	−0.47
	Tense	33	32	34	24	17	41	30.17	8.42	23.13	4.42			
	Aspect	32	32	34	24	17	41	30.00	8.37	23.13	4.42			
	Person	33	32	34	24	17	41	30.17	8.42	23.13	4.42			
✓	Verb complexity score (IS/MV-1)	1.97	2.00	2.00	2.00	2.00	2.00	1.99	0.01	1.99	0.04	−0.11	0.916	−0.03
	**Semantic measures**													
	Word count	205	241	192	233	155	289	219.17	46.05	178.50	13.04	−2.01	**0.045**	−0.54
	Duration of the narrative for the CIU analysis (sec)	249	193	352	238	81	254	227.80	88.80	100.63	14.38	−2.20	**0.028**	−0.59
✓	Number of CIU	159	154	147	133	78	143	135.67	29.65	161.63	5.71	−2.61	**0.009**	−0.70
✓	CIU% (Idea density)	77.56	63.90	76.56	57.08	50.32	49.48	62.48	12.44	90.87	5.54	−3.10	**0.002**	−0.83
✓	CIUs per minute (Idea efficiency)	49.4	47.87	25.05	33.53	57.8	33.78	41.23	12.34	98.24	15.93	−3.10	**0.002**	−0.83
	**Measures of spontaneity and fluency disruptions**													
	Repetition	2	6	0	5	0	4	2.83	2.56	0.75	1.04	−1.61	0.108	−0.43
	Revisions	5	6	8	10	5	17	8.50	4.59	2.25	2.55	−2.69	**0.007**	−0.72
	Reformulations	0	0	0	0	0	0	0.00	0.00	0.13	0.35	−0.87	0.386	−0.23
✓	Total count of disruption of spontaneity and fluency	7	12	8	15	5	21	11.33	5.96	3.13	2.90	−2.67	**0.008**	−0.71

*Gray shaded cells represent significant difference (p < 0.05) in single-subject statistics, where individual AD's score was significantly different than the HC group mean. The check marked variables are used for group comparison in this study*.

*Crawford and Howell (1998) statistical test was used to compare each AD's score with the HC group. Singlism.exe program (2002) was used to compute the statistics*.

*Bold font in p-values indicate significant difference between HC and AD groups*.

**Table 6:** Summary of the key findings across the six domains of speech and language production, and information on the proportion of AD individuals who showed similar results to the group differences.

**Variables**	**Alzheimer's Disease (AD)**		**Healthy Control (HC)**		**Between group significant difference**	**Direction of effect for AD**	**Effect size**	**Number (proportion) of AD participants showing sign difference (total N=6)**	***z*** **-value**	***p*** **-value**	**Effect size**
	**Mean**	**SD**	**Mean**	**SD**							
**Speech rate**											
Total number of words	322.00	133.43	466.00	211.98	✗				−1.420	0.156	−0.38
Words per minute	60.07	29.52	135.92	31.89	✓	decreased	Large	5 (83%)	−2.969	0.003	−0.79
**Structural and syntactic measures**											
Proportion of words in sentences	0.86	0.05	0.80	0.15	✗				−1.941	0.052	−0.52
Mean sentence length	4.26	0.64	7.68	0.82	✓	shorter	Large	6 (100%)	−3.098	0.002	−0.83
Proportion of well-formed sentences	0.79	0.13	0.95	0.06	✓	lesser	Large	2 (33%)	−2.529	0.011	−0.68
Embedding index	0.03	0.05	0.60	0.22	✓	lower	Large	6 (100%)	−3.112	0.002	−0.83
**Lexical measures**											
Proportion of noun (N/all NW)	0.33	0.04	0.33	0.03	✗				−0.713	0.476	−0.191
Proportion of pronoun (P/all NW)	0.05	0.03	0.10	0.03	✓	decreased	medium	3 (50%)	−2.274	0.023	−0.61
Proportion of pronoun to noun (P/P+N)	0.14	0.08	0.24	0.06	✓	decreased	medium	4 (67%)	−2.132	0.033	−0.57
Proportion of verb (V/all NW)	0.27	0.02	0.24	0.04	✗				−1.431	0.152	−0.382
Proportion of nonfinite verb (NF/all V)	0.21	0.10	0.38	0.07	✓	decreased	large	5 (83%)	−2.791	0.005	−0.75
Porportion of matrix verb (MV/all V)	0.79	0.10	0.62	0.07	✓	increased	large	5 (83%)	−2.726	0.006	−0.73
Proportion of compound verb (CV/all V)	0.35	0.11	0.34	0.12	✗				−0.258	0.796	−0.07
Proportion of postposition (PP/NW)	0.09	0.03	0.08	0.02	✗				−1.31	0.192	−0.35
Number of reduplication	0.50	0.55	3.00	2.78	✓	decreased	medium	3 (50%)	−1.994	0.046	−0.533
**Morphological and inflectional measures**											
***Nouns inflections***											
Noun inflection index	0.98	0.03	1.00	0.00	✗				−1.695	0.090	−0.45
Proportion of inflected nouns	60.95	14.39	58.05	10.72	✗				−0.258	0.796	−0.07
Proportion of noun with 1 inflection	0.82	0.06	0.85	0.09	✗				−0.584	0.559	−0.16
Proportion of noun with 2 or more inflections	0.17	0.06	0.18	0.07	✗				−0.390	0.697	−0.10
Proportion of definiteness markers in %	60.38	19.95	27.09	12.07	✓	increased	medium	5 (83%)	−2.453	0.014	−0.656
Proportion of case markers in %	39.16	17.18	72.44	12.56	✓	decreased	large	5 (83%)	−2.711	0.007	−0.725
***Verb inflections***											
Verb inflection index	1.00	0.00	1.00	0.00	✗				0.000	1.00	0.000
Verb complexity score	1.99	0.01	1.99	0.04	✗				−0.106	0.916	−0.028
**Semantic measures**											
Number of CIU	135.67	29.65	161.63	5.71	✓	fewer	medium	4 (67%)	−2.611	0.009	−0.70
CIU% (Idea density)	62.48	12.44	90.87	5.54	✓	decreased	large	6 (100%)	−3.102	0.002	−0.83
CIUs per minute (Idea efficiency)	41.23	12.34	98.24	15.93	✓	decreased	large	6 (100%)	−3.098	0.002	−0.83
**Measures of spontaneity and fluency disruptions**											
Total count of disruptions of fluency (repetition, revision, reformulations)	11.33	5.96	3.13	2.90	✓	greater	large	3 (50%)	−2.673	0.008	−0.71

*Gray shading indicates significant group difference*.

In terms of rate and spontaneity of speech, compared to the HC, AD individuals produced a slower rate of speech with higher number of disruptions to spontaneity and fluency of speech. Table 5 indicates that revisions caused the most common type of disruption to the spontaneity of speech. Individual level analyses revealed that slow speech rate was observed in majority of AD participants (five out of six) and disrupted spontaneity was evident for three out of six participants.

In terms of syntactic and structural features, compared to the HC, AD individuals produced shorter (smaller mean sentence length), grammatically simpler (lower embedding indexes), and less well-formed sentences. Individual level analyses revealed that shorter length and lower embedding index was present in all of our AD participants. In contrast, ill-formed sentences were observed only in two of the six participants. Some sources of ill-formedness of the sentences were: Unclear or missing subjects, despite pro-drop being allowed in Bengali; incomplete sentences; missing coordinating conjuncts; correct but overuse of a specific marker; subject, object or verb on some occasions replaced by fillers or particles. Table 3 provides illustrative examples of these errors.

In the domain of lexical measures, compared to the HC, AD individuals showed reduced proportion of pronouns, decreased proportion of nonfinite verbs, increased proportion of matrix verbs, and fewer reduplications. All other distributions and proportions of lexical items were comparable between the two groups. Individual level analyses revealed change in the proportion of pronouns (four out of six), matrix verbs (five out of six), and nonfinite verbs (five out of six) in majority of the AD participants (see Table 5).

For the morphological and inflectional measures, AD and HC participants demonstrated equivalent inflectional indices both for nouns and verbs. This implies that AD participants were able to provide correct and appropriate inflections for the nouns and verbs they produced. Further, AD participants could also produce similar proportion of inflected nouns and similar proportion of nouns with one or two inflections (see Table 5). However, contrast could be observed between the two groups in terms of the type of noun inflections: AD participants produced higher proportion of definiteness markers, whilst HC produced greater proportion of case markings. The pattern of higher proportion of definiteness markers for nouns and lower proportion of case markers were observed for five out six AD participants. AD participants did not show any difference in the inflectional complexity scores for verbs, indicating that they could produce similar quantity of inflections compared to the controls. In the domain of semantic content and CIU analyses, compared to the HC, AD individuals showed fewer CIUs, lower idea density and idea efficiency. Individual level analyses revealed every AD participant had lower idea density and efficiency (six out of six). It is worth noting that the relationship between overall dementia severity and deficits in connected speech is far from straightforward. With the exception of one AD participant, AD07, who had a dementia rating of two, all other five participants evidenced a severity rating of one (i.e., mild). Despite AD07 demonstrating more severe dementia compared to the others in the group, she did not necessarily show more severe deficits on connected speech variables.

In summary, from Table 6 we can see that the parameters which most prominently distinguished AD from the HC with large effect sizes and were impaired in majority of AD participants (at least four out of six) include: slowed speech rate; shorter sentence length; fewer embeddings; decreased proportion of pronouns; increased proportion of matrix verb with decreased proportion of non-finite verbs; decreased proportion of case marking for nouns with increased proportion of definiteness markers; and semantically reduced idea density and idea efficiency. In addition, disruption in spontaneity and fluency, decreased numbers of reduplications, and decreased proportion of well-formed sentences showed significant group differences with fewer AD participants.

## Discussion

We undertook this research to characterize connected speech production and identify linguistic features of Bengali AD participants. The impetus for this work was driven by the fact that an accumulating body of research has shown that speech and language characteristics of connected speech provide a valuable tool for identifying, diagnosing and monitoring progression in AD. However, our knowledge of linguistic features of connected speech in AD is primarily derived from English speakers. This is a problematic situation. The world is full of languages that are linguistically different from English. In fact, the majority of world's population do not speak English as their primary language. Therefore, there is an urgent need to investigate whether linguistic features that are used for characterizing AD in English will be relevant for structurally distinct languages. This is what we set out to find in speakers of Bengali, a pro-drop, Indo-Aryan language, and which is the seventh most spoken language in the world.

The key findings indicate that Bengali AD participants showed both similarities to findings reported from English speaking AD subjects as well as language specific differences from English. Similarities with English speaking literature were decreased speech rate, simplicity of sentence forms and structures, and reduced semantic content.

Critically, differences with English speakers' literature emerged in the domains of linguistic features where Bengali differs, such as pro-drop nature of the language and inflectional properties of nominal and verbal systems. Specifically, Bengali AD participants produced fewer pronouns, which is in contrast with a key feature of English AD speakers who produce an abundance of pronouns in connected speech. Despite Bengali being a highly inflected language, our AD participants showed a similar amount of noun and verb inflections without any obvious difficulties. However, differences did appear in the type of noun inflections that the AD speakers used, in most instances choosing simpler inflectional features.

Overall, connected speech production in these AD participants was characterized by the use of simpler, less complex and operationally less demanding options, with impoverished semantic content. They used shorter and simpler sentences with reduced rate of speech and reduced spontaneity, using fewer pronouns, fewer reduplications, and demonstrated a lack of difficulty with the quantity of noun and verb inflections produced but using inflections that are simpler. In the following paragraphs, we discuss the findings in detail and highlight how this research provides seminal evidence to build future research with different languages.

The finding that our AD participants produced a slower rate with higher number of disruptions to spontaneity because of revisions corroborates existing literature (Sajjadi et al., 2012; Forbes-McKay et al., 2013; Ash and Grossman, 2015). They produced significantly shorter sentences, which were grammatically simpler with minimal embeddings, and at times also fewer well-formed sentences. The majority of AD participants in our study showed difficulty with speech rate (5/6), shorter MLU (6/6), and fewer sentence embeddings (6/6) highlighting the consistency of these features across AD patients. Although poorly formed sentences showed a significant group difference, it arose from only two of the six participants (AD01, AD06). The reason for less well-formed sentences was because the sentences had missing or under specified lexical items, mostly objects or subjects but at times even verbs resulting in incomplete sentences. Recall that unlike English, Bengali allows a more flexible word order, it permits greater leeway to formulate grammatically correct and well-formed sentences. Despite this feature two of the AD participants produced significantly fewer well-formed sentences. These findings of simplified syntactic production are in concordance with AD connected speech literature (Ash et al., 2007; Cuetos et al., 2007; De Lira et al., 2011; Sajjadi et al., 2012; Ahmed et al., 2013; Forbes-McKay et al., 2013; Ash and Grossman, 2015; Fraser et al., 2016).

An interesting question arises as to why these AD participants were producing syntactically and grammatically simpler sentences. Prior AD literature suggests that participants have significant impairments in their memory processes, which contributes to their difficulty in syntactic operations (e.g., Waters et al., 1998). This could indeed be a possibility in our data as most of our participants have lower scores on background memory measures. Another contending explanation is that our AD participants demonstrated grammatical difficulty as noted by other authors (e.g., Fraser et al., 2016). Fraser et al. (2016) noted that the syntactic impairments in their AD participants' picture description had features similar to Broca's aphasia, but commented that “while these deficits resemble Broca's aphasia and progressive nonfluent aphasia in their form, they are less severe, seldom reaching the point of frank agrammatism or telegraphic speech seen in those disorders” (p. 414). The difficulty with syntax and grammar is evident in our participants if we carefully consider the lexical distribution of types of verbs in the narratives. The findings of fewer nonfinite verbs produced by the AD participants correspond to the associated lack of complexity and embedding of their sentences. However, when the embedded clauses were indeed produced, the verbs were appropriately marked for agreement. This suggests that the difficulty was is in the structural complexity of the sentence rather than in inflectional morphology. This is consistent with previous studies in languages with high inflectional morphology, in that, the inflectional morphology is spared in cases of language impairments (Leonard, 2000; Penke, 2009; Auclair-Ouellet et al., 2019). Instead, the participants with AD in our study produced shorter sentences with single matrix verbs. Individual level analyses revealed an increase in the proportion of matrix verb with a corresponding decrease in nonfinite verbs in the majority of the AD participants (see Table 5). Future research using sentence production and comprehension tasks, with different sentence types and varying syntactic complexity would be important to understand the mechanism that is underplaying in the production of syntactically simplified connected speech in AD.

In terms of lexical measures and distribution of various lexical classes, the most salient finding from this research is that Bengali speaking AD showed a reduced proportion of pronouns in their narrative samples. As a group, AD participants produced fewer pronouns; four of the six participants produced significantly fewer pronouns compared to the controls; two produced similar number of pronouns to the controls. Importantly, none of them over produced pronouns. This finding is in stark contrast with the findings from English speaking AD participants where over production of pronouns is a distinctive feature (March et al., 2006; Ahmed et al., 2013; Jarrold et al., 2014; Fraser et al., 2016). Increased production of pronouns has also been reported from AD speakers of Hebrew (Kavé and Levy, 2003; Kavé and Goral, 2016). Recall that Bengali is a pro-drop language and allows dropping of the subject; the subject could be inferred from the other inflected parts of speech. Pro-drop is more common with inflectionally rich languages, where inflectional morphology could be used to infer the referent. In languages where subjects are obligatorily spelled out, such as in English, dropping the subject is not an option. Therefore, AD individuals of those languages such as English, will prefer pronouns over nouns as the former is semantically vague, more frequent in use and thus might be easier to retrieve. In contrast, Bengali allows null-subject (i.e., dropped subject). Participants can drop the subject as null subject is cognitively less costly (Bloom, 1990). However, in English when one has to produce something, a less costly option is usually opted for, which is over-producing the pronoun (Almor et al., 1999). One simple deduction can be drawn from this cross-linguistic observation: when a language allows the avoidance of a linguistic feature or structure, such as subject drop in Bengali, AD participants will avoid it as retrieving and producing the subjects is more demanding. In contrast, when a language does not allow the avoidance of a linguistic feature, such as the obligatory use of a subject in English, AD participants will opt for a cognitively less costly option, that is, the replacement of nouns with pronouns. The important implication for this finding is that over-production of pronouns, which is a characteristic feature in English, might not be a relevant linguistic marker for a pro-drop language, such as Bengali. Research investigating pronoun usage for AD speakers in other pro-drop languages will be of great importance to determine if this pattern holds true across languages.

Reduplication is a frequent lexical feature in Bengali which is employed by speakers to enhance senses of multiplicity, continuation of action, recurrent happening of an event, or emotional state. In a sense, it serves a semantic function but requires word formation processes to generate the reduplicated forms. Using reduplication allows the expression of a richer and enhanced sense of the concept or event; however, lack of use of reduplication is not a linguistic deficit. AD participants' use of fewer reduplications could be further evidence of their difficulty in using complex linguistic operations, in this case, the word formation processes. This could indicate that AD participants have difficulty with complex word formation processes. Reduced reduplication has been reported in individuals with aphasia speaking standard Indonesian (Anjarningsih et al., 2012).

In the context of semantic content analysis—idea density and idea efficiency—reflect the ability to produce relevant content efficiently at a discourse level. Unsurprisingly, results reveal that our AD participants generated less concise information as noted by reduced idea density indicating they needed more words to convey ideas. This resulted in characteristic features of “empty speech” and “non-specificity” of discourse in AD reported in the literature (e.g., Nicholas et al., 1985). Some of these features include empty phrases (e.g., *mane hacche* “it seems”), deictic terms (e.g., *edik odik* “this side that side”, *tār pare* “then”), indefinite terms (e.g., *ekṭā* “one”, *iye* “something”), and repetitions (e.g., *eṭā ekṭā kukur… kukur* “this one is dog…dog”). Along with reduced idea density, AD participants evidenced reduced rate at which meaningful information is conveyed over time, that is, reduced idea efficiency. All of our AD participants (six out of six) showed reduced idea density and idea efficiency in their narrative samples. Reduced information content resulting in limited idea density and idea efficiency is a consistent finding across AD connected speech studies (Nicholas et al., 1985; Croisile et al., 1996; Forbes-McKay and Venneri, 2005; Sajjadi et al., 2012; Ahmed et al., 2013; Forbes-McKay et al., 2013). This highlights the fact that irrespective of the language spoken by AD participants, difficulties in conveying ideas concisely and efficiently are a pervasive difficulty as noted across various production tasks such as conversations (e.g., Dijkstra et al., 2004); picture description (e.g., Ahmed et al., 2013), and interviews (Sajjadi et al., 2012).

Studies investigating morphosyntactic characteristics of connected speech by measuring differences in inflectional properties between AD and controls have been reported from English speakers [see Auclair-Ouellet (2015) for a systematic review of inflectional morphology in primary progressive aphasia and AD]. As English is not an inflectionally rich language, it offers limited opportunity to test morphosyntactic differences between control and AD. In contrast, Bengali has a rich inflectional system for nouns and verbs. The findings from this study show that AD participants and the controls produced comparable proportion of inflected nouns, as noted by the similar noun inflection index as well as comparable proportion of nouns with one and two inflections. This highlights that AD participants were able to produce noun inflections in similar quantity to the controls. This is in contrast with findings from English speaking subjects from the literature who have been reported to have difficulties with nouns with determiners (e.g., Ahmed et al., 2013). This finding is not surprising when viewed with the lens of the literature on acquisition of morphological markers in morphologically rich languages (e.g., Penke, 2012). It has been proposed that morphologically rich and agglutinative systems generally display a greater morphological transparency compared to inflection systems where the inflection is associated with changes to the stem. As such, in these morphologically richer languages, inflectional morphology is acquired earlier in comparison to languages with sparse inflectional morphology (Bates and MacWhinney, 1987; Dressler, 2010). Therefore, in our data preservation of inflectional abilities in AD participants could be a reflection of the stability of these patterns as they might have been acquired earlier.

**Figure 1:**
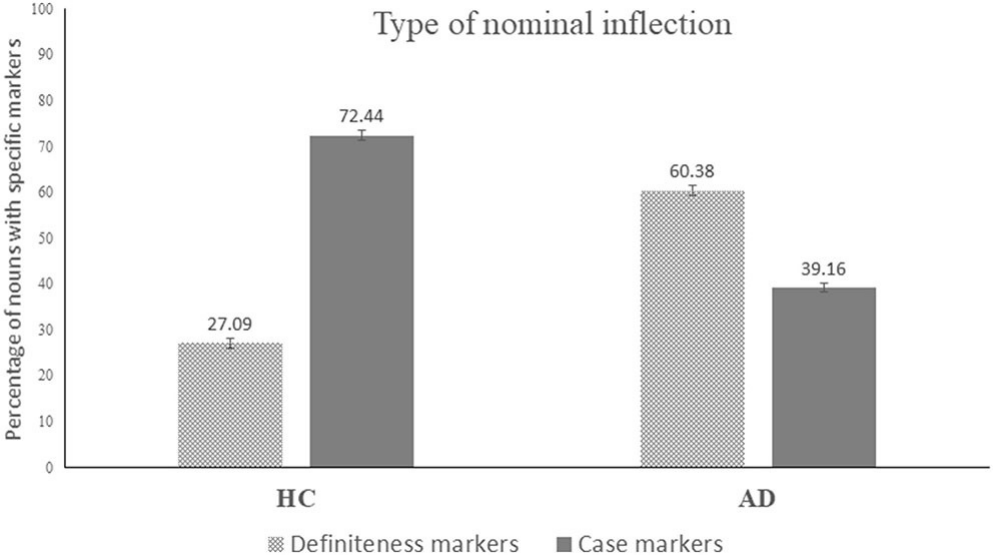
Mean proportion of nouns with definiteness vs. case markers for Alzheimer's Disease (AD) and Healthy Controls (HC). Error bars represent standard error of mean.

Distinct differences do appear between the two groups when type of noun inflections was explored in detail (see Figure 1). In AD, definiteness markers were more prevalent in nouns; whilst case marking was under-used (e.g., case-marking *jāre* “in the jar”; *kukurke* “to the dog”; definiteness marking *jāta* “the jar”; *kukurta* “the dog”). Case marking is grammatical in nature and use of appropriate case markers requires complex morphosyntactic operations. The difficulty with case marking is an indication that AD participants' difficulties in production could be in using complex grammatical operations as use of appropriate case marking requires complex morphosyntactic processes. In contrast, definiteness marker is more semantic in nature and is used as a tool for over specifying a subject or object. This finding highlights the importance of digging deeper into the morphosyntax of languages to understand the core linguistic difficulties across languages, which has the potential to inform about underlying processes as well as aid in developing specific clinical markers for diagnosis.

In terms of verbal inflections, our AD participants showed no difficulty with generating appropriate inflections for verbs, as noted by verb inflection index and verb complexity score. Any verb they produced was correctly inflected for tense, aspect, and person. Qualitatively, they produced fewer variations in these features (see illustrative examples in Table 3) but overall, they could produce correctly inflected verbs. Research from German speakers with AD (Blanken et al., 1987) and Hebrew speakers with AD (Kavé and Levy, 2003) found no difference between AD and their control groups on verb inflectional abilities. This is in contrast with the greater number of inflectional errors in English-speaking AD patients (Altmann et al., 2001; Sajjadi et al., 2012; Ahmed et al., 2013), difficulty with inflected verbs, auxiliary verbs, gerunds or participles (Fraser et al., 2016); difficulty with verb tense use (Dijkstra et al., 2004) and difficulty with subject verb agreement (Kaprinis and Stavrakaki, 2007). This is an interesting point of discussion as languages such as Bengali, German, Hebrew, who have a more complex and richer verbal inflectional system than English was not precipitating more inflectional errors in AD speakers. The answer could be found in thinking about the nature of this complexity. In these languages, the verbal inflectional system is complex but regular and systematic. That complexity does not equate to difficulty has been shown in morphologically richer languages even in child acquisition literature (e.g., Penke, 2012). As argued earlier, the complex morphological structures that are acquired earlier might have been better preserved. We believe that a future line of research which systematically compares inflectional morphology and its breakdown across different languages stands to inform our understanding of core linguistic deficits across various dementia syndromes.

## Considerations for Future Research and Limitations

In this section, we share our experiences and “lessons learnt” from embarking on connected speech research in an unexplored language, especially in determining an appropriate task and linguistic analysis framework for the data. Given that research is a resource intensive enterprise, we believe that documenting these observations would be useful for future researchers interested in similar research in neurological impairments in languages that have not yet been studied. We also highlight limitations of our current study and suggest future research directions.

First, if one is interested in characterizing linguistic patterns of connected speech in AD in a language, which has yet not been documented, the choice of task has important implications for the conclusions that could be drawn based on the findings. Picture description is quick and easy to administer. However, several studies with neurological impairments have reported that picture description often generates impoverished speech with limited types of sentence production, and patients often default to listing of the elements in the picture rather than producing “connected” speech *per se* (e.g., Olness, 2006; Armstrong et al., 2013). Interviews on the other hand are time-consuming and lack consistency across participants. It is ideal to use a linguistic task, which allows the person to generate connected speech samples with a story line (e.g., narrative story retell tasks). Grossman (2012) noted that connected speech features in dementia “are best quantified by a semi-structured protocol that is long enough to show the variety of utterances that can occur in spontaneous speech, yet is standardized enough so that all participants have an opportunity to produce speech prompted by the same content” (p. 546). The type of data generated in story narratives, such as Cinderella or Frog Story, affords opportunities to analyze connected speech both at micro- and macro-linguistic levels. It has also been suggested that it is prudent to use multiple elicitation methods in research studies to fully capture production differences across tasks (Boyle, 2015; Stark, 2019), which in turn can help decide the best task for clinical use. For our study, we used the Frog story as it allowed richer output and was culturally appropriate for our participants. Once a baseline of deficits is established in a new language using a semi-structured task, further research could be conducted to compare language production across different tasks (e.g., story narrative, picture description). Our current research focused on the micro-linguistic structures of production; macro-linguistic analysis of narratives remains a productive area of research in AD. Future research using multi-level analyses of micro-and macro-linguistic structures will further improve our understanding of connected speech profiles in AD.

Second, having an excellent team with interdisciplinary expertise is important. Critically, in-depth knowledge and understanding of linguistics of the language studied is essential. Without the linguistic expertise, it is possible to miss important features of the language that could serve as linguistic markers of the impairments. As illustrated from the current research, the differences between AD and controls in the type of nominal inflections used highlights specific linguistic differences between the two groups; whilst restricting our analysis to overall noun inflection index would not have revealed the true nature of the deficits in AD participants. Future research that aims to characterize impairments in languages that have not been studied should strive to provide an exhaustive characterization of the linguistic features as these documentations over time could lead to a greater understanding of how different languages breakdown in AD.

Third, linked with the linguistic knowledge is the choice of analysis framework. We used the well-tested multidimensional analysis system of the QPA and augmented the framework with additional measures to capture Bengali specific linguistic features, as well as semantic content analysis. We found this approach useful, as it remained in line with the analysis framework that most researchers in this field are using (Slegers et al., 2018). Using a well-established method for analyzing and reporting data that is accessible to readers in the field would be an important consideration for future researchers. This will ensure that research findings from new languages remain comprehensible for readers who are non-speakers of those languages. We are happy to discuss and share with interested researchers the steps we followed in augmenting the QPA to suit the needs for Bengali.

Fourth, although it might be obvious, we emphasize the importance of clear task instructions and well-documented administration protocol especially for testing linguistically and culturally diverse populations. For example, bilingual clients who are proficient in both languages and in their naturalistic speech might code-switch effortlessly. In these instances, it will be beneficial to mention if the testing was conducted in bilingual vs. monolingual mode and how strictly those modes were followed. The corpus of language output, its analysis and interpretation would be different when bilinguals are allowed to use bilingual mode instead of monolingual mode. Future research with bilingual clients including various modes of elicitation stands to inform language processing and language control in them, and whether bilinguals can harness the power of two languages to provide a more productive output.

Fifth, recruiting a large sample of well-controlled and well-characterized clinical group remains a perennial difficulty for researchers. For this research we had six AD participants. A larger sample of AD participants would, of course, be desirable, although such number is not unusual in clinical studies particularly where participants belong to an under-represented group. The methodology was selected to mitigate challenges of generalization. As such, statistical analysis captured findings at both the group and individual levels, offering a comprehensive, detailed and nuanced approach to the profiling of linguistic impairments in a language which has not yet been linguistically studied in depth in neurological impairments. Future research with larger sample sizes with varying severity is desirable. As seen amongst the AD participants in this research that higher overall dementia severity did not necessarily reflect most difficulties in linguistic features. We urge caution in establishing direct link with overall dementia severity to the linguistic profiles of AD participants. In addition, consorted efforts for data sharing and data deposits amongst researchers and clinicians would enable collection of larger datasets.

Sixth, it is likely that non-English speakers would come from culturally diverse populations and perhaps from non-Western countries. In such situations the challenges of undertaking cross-cultural neuropsychological and neurolinguistic research should be acknowledged with clear mention of how tasks and tools used for profiling a client are appropriate and reliable. For instance, a published version of ACE for Bengali does not yet exist. Accordingly, the adapted version was used for this research, reliably adapted at the regional center we recruited from. Moreover, the population we recruited were highly educated pre-morbidly, and most were working professionally. Therefore, we did not face the typical challenges of testing lower literacy populations. However, going forward, having protocols and training in place to ensure reliability of methods for generating quality data will be of utmost importance.

## Summary and Conclusions

In summary, in this research we characterize connected speech production in Bengali AD participants. Our research is the first of its kind to provide a comprehensive and detailed characterization of linguistic features in Bengali speaking AD individuals. Such detailed characterization in South Asian languages is currently non-existent. The findings highlight that Bengali AD participants showed both similarities to findings reported from English speaking AD subjects as well as language specific differences compared to English. Similarities with English speaking literature gravitated toward decreased speech rate, simplicity of sentence forms and structures, and reduced semantic content. Critically, differences with English speakers' literature emerged in the domains of Bengali specific linguistic features; fewer pronouns, fewer reduplications and a similar quantity of noun and verb inflections without obvious errors. Specifically, connected speech productions of Bengali AD participants were characterized by: impoverished semantic content with higher rate of disruption to spontaneity of speech and slower rate of speech; use of simpler, shorter and grammatically less complex sentences with limited embeddings; use of fewer pronouns and fewer reduplications; similar level of noun and verb inflections, but using inflections that are operationally simpler such as definiteness markers in nouns instead of case markers. This paints the picture of semantic difficulties along with differences in grammaticality of production where AD individuals choose simpler and operationally less demanding options.

This study is a significant step forward for improving both our theoretical understanding of linguistic deficits in AD and clinical implications of implementing these for improving diagnosis and monitoring progress in AD. Theoretically, this research contributes to the understanding of language impairments in neurodegenerative diseases; this could ultimately identify the core underlying impairments that result in specific linguistic profiles. The study also provides a framework for cross-linguistic comparisons across structurally distinct and under-explored languages, and also challenges the notion that more complex morphology is more difficult for AD. This research begins to address the urgent need to develop language specific linguistic markers for AD, which in turn can aid in creating clinical guidance for assessment of this community of patients in dementia services to help with sensitive and, importantly specific diagnosis of dementia disorders.

## Data Availability Statement

The raw data supporting the conclusions of this article will be made available by the authors, without undue reservation.

## Ethics Statement

The studies involving human participants were reviewed and approved by University of Reading (Ref: 2017-035-AB). The patients/participants provided their written informed consent to participate in this study.

## Author Contributions

AB, AD, and SA: conceptualization. AD and RN: participant recruitment and data collection. AB, ND, and MD: linguistic framework development. AD, RN, AB, and SA: neuropsychological data coding and interpretation. AB, ND, MD, TD, YC, and SA: linguistic data coding and analysis. AB, YC, and TD: statistical analysis. AB, ND, SA, and MD: writing and critical review. All authors contributed to the article and approved the submitted version.

## Funding

AB was supported by the Centre of Literacy and Multilingualism (CELM) pump priming grant from the University of Reading. ND was supported by the British Academy International Visiting Fellowship Grant (VF1/103620; Visiting Fellowships Programme 2018). TD was supported by the University of Reading's 2020 UROP summer studentship.

## Conflict of Interest

The authors declare that the research was conducted in the absence of any commercial or financial relationships that could be construed as a potential conflict of interest.

## Publisher's Note

All claims expressed in this article are solely those of the authors and do not necessarily represent those of their affiliated organizations, or those of the publisher, the editors and the reviewers. Any product that may be evaluated in this article, or claim that may be made by its manufacturer, is not guaranteed or endorsed by the publisher.
